# SeedSeq: Off-Target Transcriptome Database

**DOI:** 10.1155/2013/905429

**Published:** 2013-08-29

**Authors:** Shaoli Das, Suman Ghosal, Jayprokas Chakrabarti, Karol Kozak

**Affiliations:** ^1^Indian Association for the Cultivation of Science, Kolkata, West Bengal 700032, India; ^2^Gyanxet, BF 286 Salt Lake, Kolkata, West Bengal 700064, India; ^3^Medical Faculty, Technical University of Dresden, 1307 Dresden, Germany; ^4^Universitätsklinikum Dresden, Blasewitzer Straße 43, 01307 Dresden, Germany

## Abstract

Detection of potential cross-reaction between a short oligonucleotide sequence and a longer (unintended) sequence is crucial for many biological applications, such as high content screening (HCS), microarray nucleotide probes, or short interfering RNAs (siRNAs). However, owing to a tolerance for mismatches and gaps in base-pairing with target transcripts, siRNAs could have up to hundreds of potential target sequences in a genome, and some small RNAs in mammalian systems have been shown to affect the levels of many messenger RNAs (off-targets) besides their intended target transcripts (on-targets). The reference sequence (RefSeq) collection aims to provide a comprehensive, integrated, nonredundant, well-annotated set of sequences, including mRNA transcripts. We performed a detailed off-target analysis of three most commonly used kinome siRNA libraries based on the latest RefSeq version. To simplify the access to off-target transcripts, we created a SeedSeq database, a new unique format to store off-target information.

## 1. Introduction

**Figure 1:**
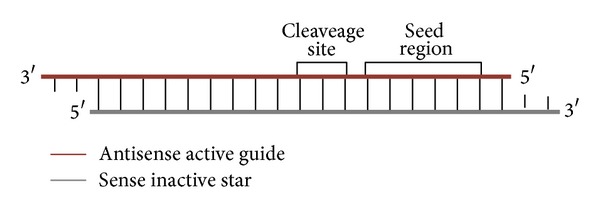
Structure of an siRNA: 21 bp RNA duplex with 2 nucleotides 3′′ overhanging on each strand; the two strands are called antisense or active or guide strand and sense or inactive or star strand, respectively; the first 2–8 bases of the antisense strand are called seed region, and at bases 8–10 of the antisense siRNA strand is the cleavage site.

Recently, RNA interference (RNAi), a natural mechanism for gene silencing, [1, 2] has made its way as a widely used method in molecular and cell biology in both academics and industry. Pharmaceutical and biotech companies have set up libraries for large-scale screens employing thousands of short-interfering RNAs (siRNAs) or short hairpin RNA (shRNA) encoding vectors to identify new factors involved in the molecular pathways of diseases [3]. The design of RNAi reagents is the key to obtaining reliable screening results in large-scale RNAi studies. Several recent studies demonstrated that the degradation of intended transcripts by siRNA (so-called “on-targets”) and unintended effects arising from inadvertent targets (so-called “off-targets”) depend on the sequence of the RNAi reagent and have to be computationally analyzed [4, 5]. For knock-down/screening purposes, different companies offer sets of siRNAs targeting the whole genome (or a subset of it) for various organisms. Typically, they offer at least three different siRNAs, for each target gene. These siRNAs can either be used as single siRNAs or can be mixed and used as a pool of siRNAs. The main reason for offering several siRNAs per target is the varying knock-down efficiency of the individual oligos and the occurrence of off-target effects. In our study, we focus on sequence-dependent off-target effects that can be attributed to the binding of the siRNA to other mRNA transcripts than their target mRNA [6, 7]. Partial complementarity between siRNA and mRNA seems to be sufficient to reduce the number of silenced mRNA [6]. Based on this tolerance for mismatches and gaps in base pairing with targets, siRNAs could have up to hundreds of potential target sequences in the genome. Currently, the degree of complementarity between the two sequences needed for silencing is not well defined. Sequence dependent off-target effects are caused in many possible ways (Table 1). First of all, it has been reported that off-target effects occur with a high probability, if the siRNA shows ~90% complementarity (17 nucleotides out of 19) to an off-target gene [8–10]. However, a 21-nucleotide double-stranded RNA sharing only partial complementarity with an mRNA is still competent to cause gene silencing via translational repression [8, 11]. It seems that as few as 11 contiguous complementary nucleotides or a total of 15 are sufficient to reduce the level of mRNA transcripts [12]. The complementarity of the siRNA seed region (the first 2–8 bases of the antisense siRNA-strand) plays a major role in the occurrences of off-target effects [9] (see Figure 1). Further analyses showed a high tolerance for mismatches outside the seed region, whereas differences within this 5′′ end of the siRNA are barely tolerated [12–14].

The center region of the siRNA is important to stabilize the siRNA-mRNA-duplex and to enhance mRNA degradation [11]. Alemán and colleagues analyzed this central region, which comprises the cleavage site of the mRNA (position 8–10 of the antisense strand; see Figure 1). They deciphered that mismatches in this region of the siRNA seem to be critical [16] and result in no cleavage. Additionally, they also tested the aspect of a G:U wobble and discovered that the G:U base pair is recognized like an authentic Watson-Crick base pair in the antisense RNA-mRNA duplex. This wobble base pairing expands the range of potential targets for a specific siRNA.

Design and validation of siRNAs are based on sequence-dependent analysis (so-called “on-target analysis”). In design process, using the sequence information, all siRNA constructs are computationally mapped onto RNA transcript sequence RefSeq-RNA using homology search algorithms. RefSeq database is a collection of taxonomically diverse, nonredundant, and richly annotated sequences representing naturally occurring molecules of DNA, RNA, and protein. Included are sequences from plasmids, organelles, viruses, archaea, bacteria, and eukaryotes. Each RefSeq is constructed wholly from sequence data submitted to the International Nucleotide Sequence Database Collaboration (INSDC). RefSeqs provide a foundation for uniting sequence data with genetic and functional information. They are generated to provide reference standards for multiple purposes ranging from genome annotation to reporting locations of sequence variation in RNAi experiments.

In order to predict off-target effects and annotate transcripts with potential off-targeting (by oligos from available siRNA libraries) information based on latest RefSeq version, a number of sequence similarity search methods or algorithms can be applied. For example, the Basic Local Alignment Search Tool (BLAST) [17] is adopted to find nearly exact homologies. Although BLAST is an excellent tool for broad sequence alignments, it falls short in its ability to accurately predict small local homologies. Other bioinformatics tools, [15, 16, 18] which do not have this shortcoming, try to predict interactions between siRNAs and mRNAs. But unfortunately, these sequence-based prediction tools frequently do not consider specific off-target parameters like target site location, 3′′UTR conserved regions, and design specificity. Also there is no standard format which allows for distribution of off-target analysis results. Here, we describe a novel method and a database, supporting the analysis of potential off-target transcripts. We will demonstrate our approach based on three available siRNA libraries. Our analysis enabled us to determine potential off-target transcripts and to create new database format called “SeedSeq.” SeedSeq aim is to provide a data source for better design, validation of siRNA libraries, and experiments. SeedSeq is available similar to RefSeq in standard gene bank [19] format file, providing easy access for bioinformatics community. SeedSeq version 1 is limited to 3 kinome libraries. Following versions will consider genome wide off-target analysis results.

## 2. Results

As we described above microRNA (miRNA)-mediated gene modulation has shown that complementary base pairing between the seed region and sequences in the 3′UTR of mRNA is associated with miRNA-mediated gene knockdown [20]. As siRNAs and miRNAs are believed to share some portion of the RNAi machinery, we investigated whether complementarity between the seed region of the siRNA and any region of the transcript was associated with off-targeting. To accomplish this, we predicted off-targets for 3 kinome siRNAs libraries (Ambion designed in 2006, Thermo Scientific Dharmacon designed in 2009; Qiagen designed in 2008). We validated prediction of off-targets using the above described method with two rounds of validation. Firstly, we validated our predictions on the siRNA transfection data from Jackson et al. [9]. Secondly, our prediction was validated against validated target sets of miRNAs that share the same seed region sequence with the siRNAs in our dataset.

**Figure 2:**
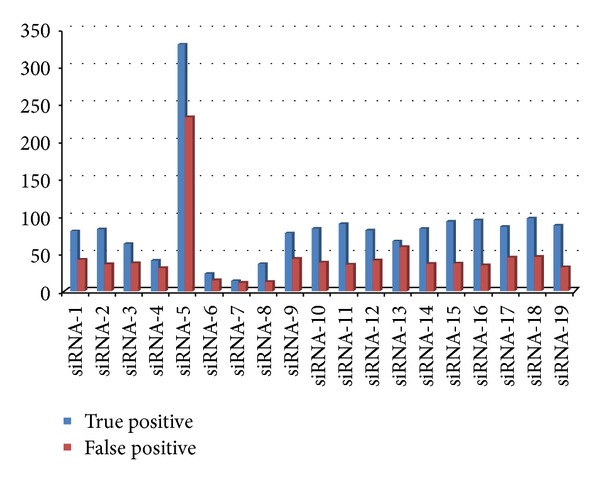
The number of true positive versus false positive off-target predictions by our algorithm for 19 siRNAs from the Jackson set. The off-target transcripts showing significant (*P* > 0.01) expression change after 24 hours of siRNA transfection were treated as true positives while transcripts not showing significant alteration in expression were treated as false positives.

From the Jackson dataset, we used the expression data after 24 hours of transfection in all the cases, and the transcripts with a negative expression change (*P* < 0.01) was regarded as off-targets. We predicted siRNA off-targets for all the 8 + 19 = 27 siRNA sequences in the dataset with/without filtering for conserved target sites. Without using conservation check, 65% of the predicted off-targets had a significantly negative expression change relative to the mock transfection control while using the filtering for conserved target sites marginally improved the true off-target prediction rate (66.85%). This again shows that target site conservation is not a significant criterion for siRNA mediated off-targeting. The true positive versus false positive off-target prediction by our algorithm for transcripts targeted by 19 siRNAs in Jackson dataset is presented in Figure 2.

For another validation, the validated set of miRNA targets are collected from Tarbase [21] and miRecords database [22]. We considered miRNAs from these databases which contain same seed sequence as siRNA and were trying to find an overlap between predicted off-targets of the siRNA and validated targets of miRNAs.

**Figure 3:**
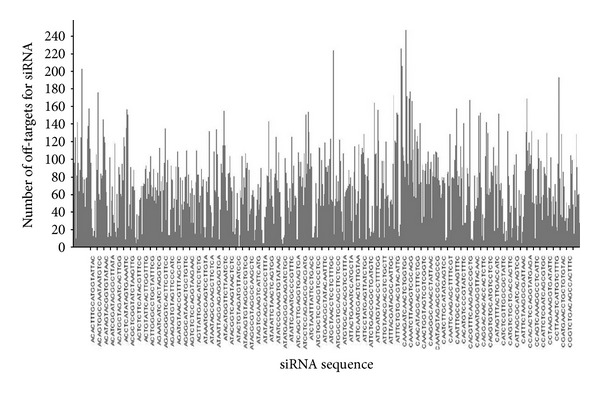
Distribution of siRNA off-targets across Dharmacon-Kinome library.

**Figure 4:**
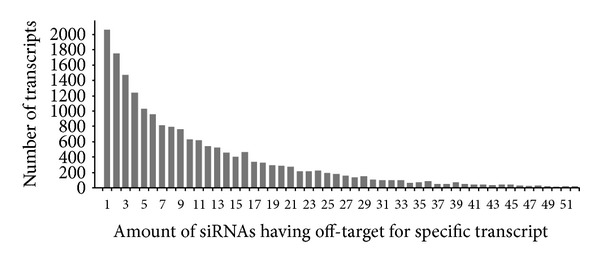
Distribution of number of transcripts versus number of siRNAs having those transcripts as off-targets based on analysis across 3 kinome libraries. For example transcript NM_020931 of gene KIAA1586 (Gene ID: 57691) is off-target of 7 ambion siRNAs: s224548, s14545, s29915, s3587, s8669, s25384, and s36140.

As a result of this validation, we found 11 siRNAs (Table 2) for which the off-target transcripts are validated targets of miRNAs with same seed sequence (4 from TarBase version 5 and 7 from miRecords), when the off-targets were predicted without conservation check. Here, we introduce a result from siRNA off-target analysis which takes into account existing kinome wide RNAi libraries using computational pipeline described above. Our analysis of three kinome-wide RNAi libraries for human revealed differences in genome coverage and off-target predicted quality. The differences most likely depend on two factors: the quality of the underlying genome release and the factors known to influence the reagent quality at the time of the library design. It has been reported that the amount of off-targets target is different for each gene and heterogeneously distributed (Figures 3 and 4). Our report shows that the amount of off-targets ranges from 0 to 240.

**Figure 5:**
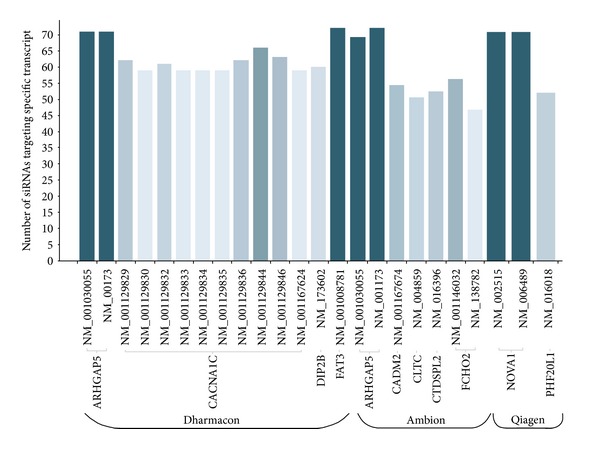
Highest number of siRNAs having off-targets for specific gene transcripts in 3 inome libraries. Transcripts of gene ARHGAP5 are the most sensitive in Ambion and Dharmacon for being an off-target transcript.

We next sought to compare 3 libraries based on which transcripts are the most sensitive transcripts targeted by siRNAs from those libraries. From 3 libraries we were able to collect the most off-target sensitive transcripts (Figure 5). Very interesting phenomenon is that both transcripts NM_001030055 and NM_001173 of gene ARHGAP5 are the most sensitive off-target transcripts in both libraries, Ambion and Dharmacon, but are targeted by different siRNAs. Frequent false positives siRNAs complicate the analysis of genome-wide RNAi screens that is why it is important to identify the candidate off-targeted transcripts in primary screening data. Several transcripts can be particularly susceptible to off-target silencing [13, 15, 18]. Such “off-targeted” transcripts are detected after much effort has been expended to validate genes of interest.

**Figure 6:**
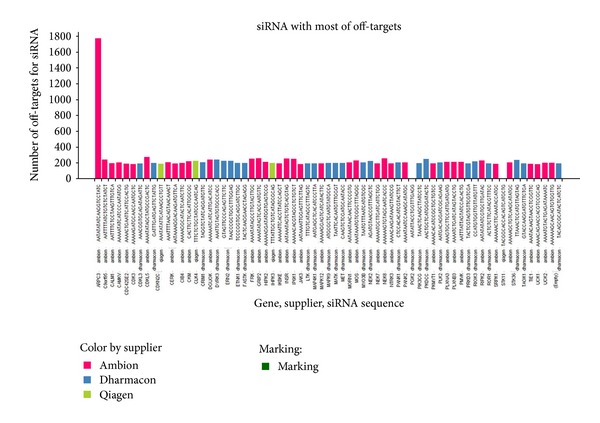
siRNAs having the highest number of off-targets across 3 libraries. siRNA designed for gene ARPC3 from Ambion is an outlier having 1800 off-targets. siRNAs designed by Dharmacon and Qiagen for gene CDKN2C indicate equal amounts of off-targets.

Our next goals were (1) to compare siRNAs with maximum number of off-targets across 3 libraries and (2) to compare transcript across 3 libraries. The distribution of siRNAs off-targets per library is not flat; some siRNAs have a large number of off-targets while many contain only a few. The distribution of the efficacies of the siRNAs is shown in Figure 6. Among the 3 libraries, Qiagen siRNAs showed lowest number of off-targets. There are only 3 siRNAs from Qiagen in the list of siRNAs from all 3 libraries with highest number of off-target transcripts. Some siRNAs particularly have many off-targets. siRNA from Qiagen designed for CDKN2C is an outlier among all siRNAs having 1800 potential off-targets.

**Figure 7:**
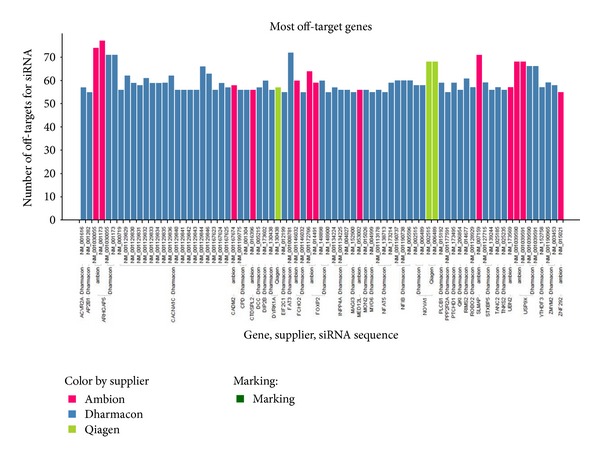
Transcripts distribution for being the most off-targeted genes across 3 libraries. For example transcripts USP9X are off-targets of oligos from two libraries parallely. Those transcripts indicate high sensitivity for being off-targets. All transcripts of gene CACNA1 are shown to be off-target sensitive.

**Figure 8:**
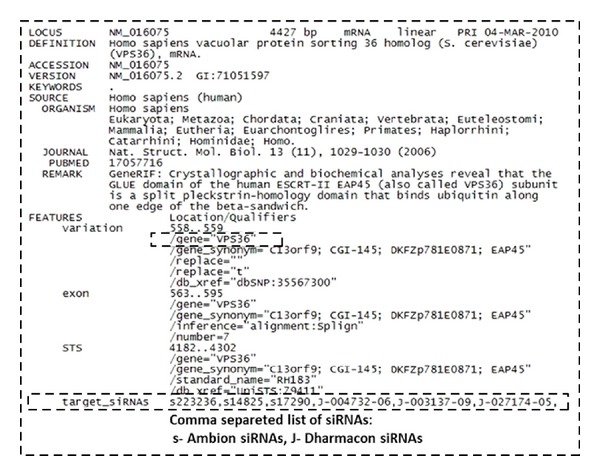
SeedSeq record example.

We selected the genes having the most off-targeted transcripts and made a comparison across the 3 libraries (Figure 7).  Figure 8 shows that it is possible to have gene transcripts which are unintentionally targeted by around 70 siRNAs parallel in two libraries (e.g., gene USP9X has two transcripts which are off-targets of ~70 siRNAs Ambion, ~70 siRNAs Dharmacon). We also selected gene CACNA1 whose all transcripts are highly off-target sensitive.

## 3. Discussion

Off-target transcripts analysis results can enhance the validation rate in RNAi screens. To our knowledge, today SeedSeq is the new format and unique collection of human mRNA transcripts and their siRNA reagents off-target predictions available in GenBank format. It was designed to assist experimentalists in determining which transcripts are the sensitive for being off-target. Our analysis revealed a record of prominent off-targeted transcripts for several available siRNAs libraries. It is not clear why several transcripts behave similar to miRNA-like off-target effects. SeedSeq may be used in the siRNA design algorithms. Usage of our method as an additional analysis component of RNAi cell based screens should enable researchers to counter-screen for downregulation of sensitive transcripts and reduce the false positive siRNAs during the validation process. Detection of transcripts sensitive to off-target effects will also enable a better understanding of the rules like miRNA-like off-targeting and improve the design quality of siRNA reagents.

## 4. Material and Methods

### 4.1. SeedSeq Format

The Seed Sequence (SeedSeq) database is an open access (RNAiAtlas http://rnaiatlas.ethz.ch/), annotated and curated collection of publicly available mRNA transcripts and their siRNAs targeting them as off-target. This database is built based on off-target analysis described below, and, like RefSeq, provides only a single record for each mRNA transcript of natural biological molecule (i.e., DNA, RNA) at the current stage for 3 kinome siRNA libraries. It is a unique resource because it provides a curated sequence database linked records from siRNA to target transcripts.

For each target gene, SeedSeq aims to provide the gene transcripts and the information about siRNAs (siRNA supplier identifier, siRNAID) which potentially target those transcripts. SeedSeq is provided in GenBank format and is currently limited to kinases. Transcripts sequence records are presented in a standard format and subjected to computational validation. SeedSeq similar to RefSeq contains different transcripts categories: NM-mRNA, NR-ncRNA, XM-predicted mRNA model, and XR-predicted ncRNA model. SeedSeq is accessible via BLAST, Entrez readers, and RNAiAtlas site. SeedSeq records appear similar in format to RefSeq and GenBank records. A sequence in GenBank sequence format is a rich format for storing sequences and associated annotations. It shares a feature table vocabulary and format with the EMBL and DDJB formats. An example sequence in GenBank format is

 LOCUS GXP_170357 743 bp DNA
 DEFINITION loc=GXL_141619*|*sym=TPH2*|*geneid=121278*|*acc=GXP
   _170357*|*  taxid=9606*|*spec=Homo
   sapiens*|*chr=12*|*ctg=NC_000012*|*str=(+)*|*
   start=70618393*|*end=70619135*|*len=743*|*tss=501,632*|*
   homgroup=4612*|*promset=1*|*descr=tryptophan hydroxylase 2*|*
   comm=GXT_2756574/AK094614/632/gold;
   GXT_2799672/NM_173353/501/bronze
 ACCESSION GXP_170357
 BASE COUNT 216 a 180 c 147 g 200 t
 ORIGIN
   1 TTGATTACCT TATTTGATCA TTACACATTG TACGCTTGTG TCAAAATATC ACATGTGCCT
   61 TATAAATGTG TACAACTATT AGTTATCCAT AAAAATTAAA AATTAAAAAA TCCGTAAAAT
   121 GGTTTAAGCA TTCAGCAGTG CTGATCTTTC TTAAATTATT TTTCTAATTT TGGAAAGAAA
   181 GCACAAAATC TTTGAATTCA CAATTGCTTA AAGACTGAGG TTAACTTGCC AGTGGCAGGC
   241 TTGAGAGATG AGAGAACTAA CGTCAGAGGA TAGATGGTTT CTTGTACAAA TAACACCCCC
   301 TTATGTATTG TTCTCCACCA CCCCCGCCCA AAAAGCTACT CGACCTATGA AACAAATCAC
   361 ACTATGAGCA CAGATAACCC CAGGCTTCAG GTCTGTAATC TGACTGTGGC CATCGGCAAC
   421 CAGAAATGAG TTTCTTTCTA ATCAGTCTTG CATCAGTCTC CAGTCATTCA TATAAAGGAG
   481 CCCGGGGATG GGAGGATTCG CATTGCTCTT CAGCACCAGG GTTCTGGACA GCGCCCCAAG
   541 CAGGCAGCTG ATCGCACGCC CCTTCCTCTC AATCTCCGCC AGCGCTGCTA CTGCCCCTCT
   601 AGTACCCCCT GCTGCAGAGA AAGAATATTA CACCGGGATC CATGCAGCCA GCAATGATGA
   661 TGTTTTCCAG TAAATACTGG GCACGGAGAG GGTTTTCCCT GGATTCAGCA GTGCCCGAAG
   721 AGCATCAGCT ACTTGGCAGC TCA.

The format also allows for sequence names and comments to precede the sequences. Attributes in FEATURE section novel to SeedSeq records include unique information about siRNAs which potentially target this record transcript. Off-target information is described as feature annotations. This annotation is provided by off-target analysis. SeedSeq record may be an essentially unchanged, validated copy of the original RefSeq record extended with off-target features or includes additional information supplied by siRNA on-target analysis. The GenBank format originates from the GenBank software package but has now become a standard in the field of bioinformatics. The simplicity of GenBank format makes it easy to manipulate and parse sequences using text-processing tools and languages like Java, Python, Ruby, and Perl. A FEATURES section (Figure 8) is designed to be associated with a sequence and can have a location on that sequence. It is a way of describing the characteristics of a specific part of a sequence. siRNA off-target information in SeedSeq is saved in FEATURE section under tag “target_siRNAs.” FEATURES are in programming languages represented by SeqFeature objects. SeqFeature objects can also have one or more annotations associated with them. SeedSeq file can be accessed similarly as GenBank, SwissProt, or EMBL file using standard GenBank format readers. An example is a SeqIOTool25 class which contains methods for reading GenBank, SwissProt, and EMBL files.

### 4.2. Off-Target Analysis Concept

Available sequence analysis tools fail to reliably predict off-target transcripts for siRNA sequences. Building upon current understanding for the occurrence of off-target effects, a new modular analytic process is applied to create SeedSeq database. This process can be specifically adapted to a variety of options in results interpretation to identify potential off-target transcript for every siRNA of interest.

### 4.3. The Analytic Process

**Figure 9:**
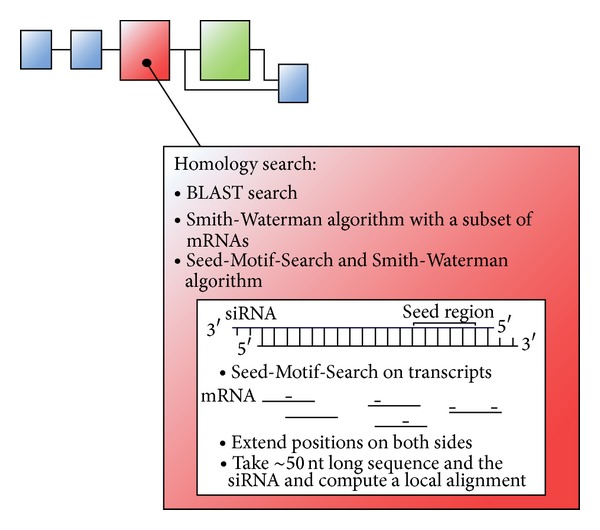
General structure of the concept for analysing screening results of off-target effects. Three variants of complementarity search for finding potential off-target effect depending on the type of off-targets.

Potential off-target effects are predicted based on sequence complementarity regions between siRNAs and mRNAs. For flexibility and extensibility reasons, the process is composed of a set of steps, which must be performed in sequence to get to an effective analysis (see Figure 9). 

The first step is to find homologies between siRNAs and all mRNAs available in RefSeq database. This concept contains many variants for such a complementarity search using different algorithms to perform a sequence alignment between siRNA and mRNA. A detailed description of the different complementarity search strategies is given in the next subsection.

The resulting list of a complementarity search can be too long to find the important results just by visual inspection. Therefore, the next step is to filter this list to reduce its size to meaningful results.

#### 4.3.1. Complementarity Search

In this analysis step, it can be determined if there exists a complementary region between the selected siRNA sequences and the mRNA transcripts. Many different sequence alignment algorithms are available to perform such a complementarity search, but they are not optimal for the purpose of this process step by default. Therefore, alternative strategy for the use of these algorithms has been developed to find nearly exact complementary regions as well as small local complementarities (see also Figure 9). 

#### 4.3.2. Smith-Waterman Algorithm

The Smith-Waterman algorithm is an accurate algorithm used to build local alignments between two sequences. Since its use with all mRNAs from the RefSeq database is not practicable, a feasible alternative is to limit the number of mRNAs to approximately 200. On a Windows 7 system (two Intel Xeon Quad-core 2.00 GHz CPUs, 16 GB RAM), the analysis of a 20-gene (4 oligonucleotides) library constructs took about 1 hour. By reducing the number of sequences, it is possible to perform a local alignment for all the siRNAs.

#### 4.3.3. Seed-Motif-Search Combined with the Smith-Waterman Algorithm

Because of the mentioned runtime problem when performing a local alignment with the Smith-Waterman algorithm, a feasible variant to search for complementarity is introduced here. In this variant, an initial step reduces the length of the mRNA sequences to enable the use of a local alignment algorithm. This reduction is made because the seed region of the siRNA seems to play a significant role in causing off-target effects. At the beginning, all occurrences of the seed motif of every siRNA are localized in the genes. After detecting this small region, a sequence of ~50 nt around this seed motif is cut out in the mRNA. Thus, as a result of this first step, a huge number of sequences of ~50 nt in length are obtained containing the seed region of each siRNA. Due to the small length of the sequences, it is now possible to perform a local alignment with the Smith-Waterman algorithm.

#### 4.3.4. miRNA-Like Off-Target Prediction

Prediction of miRNA like off-targets involves finding the seed complementarity of the siRNA with the 3′′UTR of a nontarget mRNA. But considering only seed region complementarity identifies a large number of off-targets that could not be actually targeted. For example, for haxamer siRNA seed sequences (2−7th base from 5′′ end of antisense strand), there may be thousands (or more) predicted matches in the 3′UTRs of human mRNA transcripts [10]. So, a more rational approach is needed for prediction of siRNA off-targets that needs understanding of the miRNA target recognition procedure. The Whitehead Institute siRNA selection tool uses target site conservation information for filtering the most probable off-targets. For endogenous miRNA target prediction, use of target site conservation among closely related species proved to be effective for recognition of functional target sites. For siRNA off-target prediction, restricting the seed-matched site search to the sites conserved within orthologous locations of closely related species (human, mouse, rat, and dog) greatly reduces the number of predicted off-targets and also possibly increase the chance of predicting functional target sites. For prediction of miRNA-like off-targets, we used an algorithm that combines the conventional 6-mer or 7-mer seed motif search with 3′ compensatory rule of miRNA target prediction. To check if target site conservation is a considerable factor in predicting siRNA off-targets, we searched for sites conserved among human, chimp, mouse, rat and dog.

*Seed Motif Search*. We used Smith-Waterman algorithm for alignment of the siRNA sense strand with the target mRNA. For the optimal alignment output, we considered the targets that have (1) perfect match with the nucleotides 2–8 (for 7-mer (m8) seed) from the 3′′ end of the siRNA sense strand and (2) one mismatch in the above said seed region but perfect match with the nucleotides 13–19 from the 3′′ end of the siRNA sense strand (3′′ compensatory rule).

*Generation of Conservation Data*. Genome wide conservation data generated using multi 46-way alignment (for 46 vertebrate species) was downloaded from UCSC genome browser. Genomic regions (within human genome) conserved within human, chimp, mouse, rat, and dog are then extracted and mapped within the coordinates of human mRNAs (downloaded from UCSC genome browser) to get the location of the conserved regions within human mRNAs. Conserved regions of length 8 bases or more are only considered.

#### 4.3.5. Dataset for Validation of Predicted Off-Targets

Dataset for off-target transcript expression change after siRNA transfection was collected from the study of Jackson et al. [7] The Jackson dataset consists of mRNA expression change data after siRNA transfection on HeLa cells for 8 different siRNAs designed for 8 different genes and 19 sequence variants of a single siRNA designed for the gene MAPK14 (GSE5814). The data included genes that displayed a significant (*P* < 0.01) difference in expression level relative to mock transfection control.

Experimentally validated miRNA targets dataset in human was collected from TarBase and miRecords. TarBase and mRecords both store manually curated collection of experimentally tested miRNA targets in human, mouse, fruitfly, worm and zebrafish. The miRNA-targets those are tested positive or negative are marked to distinguish between them. Each positive target site is described by the miRNA that binds it, the gene in which it occurs, the nature of the experiments that were conducted to test it, the sufficiency of the site to induce translational repression and/or cleavage, and the paper from which all these data were extracted.

## Figures and Tables

**Table 1:** Cause for sequence-dependent off-target effects.

#	Causes for off-target effects	References
1	Nearly exact complementarity	[8, 10]
2	15 nt in total or even 11 continuous nt match	[8]
3	Seed region complementarity	[9, 12, 15]
4	miRNA function (seed region-3*″*UTR conserved region complementarity)	[13]
5	Multiple occurrences of the seed region in an mRNA sequence	[13, 15]
6	Complementary region at the cleavage site, center of the siRNA	[11, 16]
7	Tolerance of G:U wobble	[16]
8	Seed complementation frequency	[12]
9	High G/C content in the seed region	[15]

**Table 2:** Selected siRNAs and their off-target overlap with on-targets of miRNAs from tarbase.

On-target gene symbol	siRNA antisense strand	siRNA id	Off-target gene symbol	Target of endogenous miRNA
WNK3	AAAUACUGACAAACGUGAGGC	s35278	ZEB1/TCF8	hsa-miR-200b
STRADB	AAAUACUGAUAUCCAAUGGGC	s30875	ZEB1/TCF8	hsa-miR-200b
MAPK4	UAAUGCUGAUCAACGAUCCUU	SI00606011	BACH1	hsa-miR-155
MAPK4	UAAUGCUGAUC AACGAUCCUU	SI00606011	TP53INP1	hsa-miR-155
WNK3	AAAUACUGACAAACGUGAGGC	s35278	RERE	hsa-miR-429
PLXNA3	AAAGUGCUUCCAUUGAUGAUG	s30979	Cyclin D2	hsa-miR-302b
PLXNA3	AAAGUGCUUCCAUUGAUGAUG	s30979	MBNL2	hsa-miR-302d
PLXNA3	AAAGUGCUUCCAUUGAUGAUG	s30979	VEGF	hsa-miR-372
PLXNA3	AAAGUGCUUCCAUUGAUGAUG	s30979	APP	hsa-miR-520c-3p
TYRO3	UUGGCACUAAAGGUCACCGUU	SI00288344	Mitf	hsa-miR-96
ITPKB	UUGGUCCAUAGUCUCCCUCUG	SI04435592	KCNQ1	hsa-miR-133a
TNK1	UAAUGCUCCAGGAUGCGCCAG	SI03649674	MEIS1	hsa-miR-155
PACSIN1	UUGGUCCCUCAGAUGGGCCUG	SI00127918	Pitx3	hsa-miR-133b

## List of Abbreviations

siRNA: Small interfering RNA
HCS: High content screening
RNAi: RNA interference
shRNA: Short hairpin RNA
INSDC: International Nucleotide Sequence Database Collaboration
BLAST: Basic Local Alignment Search Tool
miRNA: MicroRNA.
